# Efficiency of large-scale aided phytostabilization in a mining pond

**DOI:** 10.1007/s10653-023-01520-z

**Published:** 2023-03-09

**Authors:** Vajihe Shahrokh, Silvia Martínez-Martínez, Ángel Faz, Raúl Zornoza, Jose A. Acosta

**Affiliations:** Department of Agricultural Engineering, Technical University of Cartagena, Paseo Alfonso XIII 48, 30203 Cartagena, Spain

**Keywords:** Phytoremediation, Bioavailable metals, Vegetation cover percentage, Amendments

## Abstract

Mining activities accumulate large quantities of waste in tailing ponds, which results in several environmental impacts. In Cartagena–La Unión mining district (SE Spain), a field experiment was carried out in a tailing pond to evaluate the effect of aided phytostabilization on reducing the bioavailability of zinc (Zn), lead (Pb), copper (Cu) and cadmium (Cd) and enhancing soil quality. Nine native plant species were planted, and pig manure and slurry along with marble waste were used as amendments. After 3 years, the vegetation developed heterogeneously on the pond surface. In order to evaluate the factors affecting this inequality, four areas with different VC and an area without treatment (control area) were sampled. Soil physicochemical properties, total, bioavailable and soluble metals, and metal sequential extraction were determined. Results revealed that pH, organic carbon, calcium carbonate equivalent and total nitrogen increased after the aided phytostabilization, while electrical conductivity, total sulfur and bioavailable metals significantly decreased. In addition, results indicated that differences in VC among sampled areas were mainly owing to differences in pH, EC and concentration of soluble metals, which in turn were modified by the effect of non-restored areas on close restored areas after heavy rains due to a lower elevation of the restored areas compared to the unrestored ones. Therefore, to achieve the most favorable and sustainable long-term results of aided phytostabilization, along with plant species and amendments, micro-topography should be also taken into consideration, which causes different soil characteristics and thus different plant growth and survival.

## Introduction

Mining activities spread many inorganic and organic contaminants in ecosystems (Karbowska, 2016). Several contaminants from adjacent mining districts can be leached into soils, surface and groundwater and subsequently transferred to the food web. In addition, mine tailings characterized by null vegetation, poor fertility, low pH, high instability and high contents of potentially toxic elements (Conesa et al., 2006) may result in massive environmental degradation, erosion, air pollution, biodiversity and functionality loss and health risks to human populations in the surrounding regions (Confalonieri et al., 2014). Considering all those facts, providing an effective remediation method to rehabilitate the tailing ponds has been a great challenge in recent decades.

Different physical, chemical and biological approaches have been applied to remediate soil and mining wastes rich in potentially toxic elements and inorganic compounds (Wuana & Okieimen, 2011). Among the different approaches with their own advantages and disadvantages, special attention is drawn to the phytoremediation technologies.

Aided phytostabilization as an eco-friendly, efficient, cost-effective, simple, and applicable in long term deals with the reduction in mobility and bioavailability of potentially toxic elements in soils. This technique requires the incorporation of the vegetation with appropriate plant species and, in some cases, the adequate application of amendments (Yang et al., 2016).

The selected plant species for phytostabilization are preferred to be native as they are adapted to the local climatic and environmental conditions. The selected plants are also characterized by the fast growth, extensive root system development, large amount of biomass and tolerance to metal contamination and nutrient deficiency (Costa et al., 2016; A. L. Fernando et al., 2018a, 2018b). Among the hyperaccumulator plants, those accumulate potentially toxic elements in their below ground parts are the most effective species for phytostabilization, owing to the accumulation of metals in root tissues, metal sorption onto root surfaces and thus inhibition of metal release into the food web (Mahar et al., 2015).

Different inorganic and organic amendments can be used in abandoned soils and mine sites to achieve the optimum conditions for plant growth by improving the biological, physical and chemical properties of soil and also to stabilize metal(loid) in soil (Acosta et al., 2011; Park et al., 2011; Zornoza et al., 2013). Inorganic materials rich in carbonate are successfully applied to reduce the acidic conditions of several acid-forming mine wastes (Barker, 1997). Moreover, organic residues can significantly improve the organic matter content and fertility level; increase microbial populations or activities and possibly adsorb the metals on solid surfaces through complexation with humic substances (Ye et al., 2002).

It is well known that improvement of soil health and plant growth can be two major indicators of the effectiveness of reclamation methods and reduction of environmental risks (Epelde et al., 2009). In this regard, we applied organic and inorganic amendments in a tailing pond to improve soil conditions for establishment of native species. However, after 3 years of the amendments application and planting, the vegetation developed heterogeneously on the surface of the tailing pond. In addition, topography as one of the soil evolution factors can creates sites of variable hydrological processes, soil water availability, soil characteristics and vegetation (Biswas, 2019). Furthermore, considering the influence of microtopography on landscape properties such as soil biogeochemistry and soil surface roughness and relief (Cross et al., 2021) raises this question that whether this factor could affect the efficacy of phytostabilization of tailings ponds. Therefore, the objectives of this study were: (i) to evaluate changes in the soil characteristics after application of amendments and planting and (ii) to elucidate the factors including microtopography affecting the effectiveness of aided phytostabilization technique in a tailing pond with high concentration of Cd, Cu, Zn and Pb.

## Materials and methods

### Study area

Mine tailings are located in Cartagena-La Unión Mining District (Murcia Province, SE Spain), where great mining activities had been carried out for more than 2500 years in search for silver lead, zinc or iron (Alcolea-Rubio, 2015), stopping its activity in 1991. The mining districts are located on the eastern side of the Cordillera Bética and are part of a wide volcano-tectonic and metallogenetic belt that extends from Cabo de Gata to the Sierra de Cartagena. Mineral paragenesis has greenalite, chlorite, magnetite, pyrite, sphalerite, galena, siderite and quartz (Manteca & Ovejero, 1992). As a consequence of the long period of mining activity, large volumes of wastes were generated during the mineral concentration and smelting processes. Historically, these wastes were dumped into the streams, contaminating their surroundings. In order to remediate this situation, the Government of Spain in 1955 prohibited mining companies from dumping wastes on the ground to form tailing ponds (Vilar et al., 1991). Currently 85 tailing ponds remain in the area, which are often composed of very high concentration of metals (Martinez-Martinez et al., 2013). In addition, when high-intensity storm events occur in the area, acidic mine drainage (AMD) is generated that can contaminate the areas near the mining ponds, such as agricultural or urban areas.

**Fig. 1:**
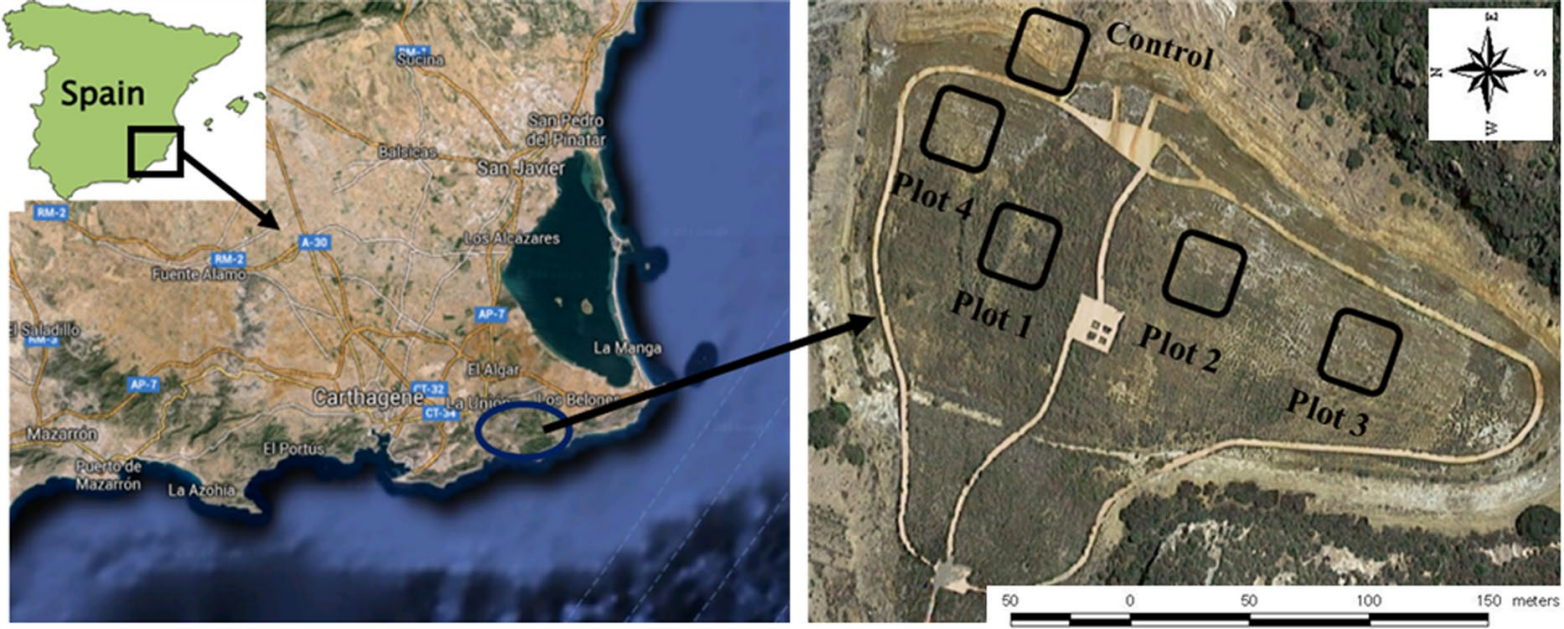
Location of the study area and view of tailing pond

The study was conducted in a tailings pond of the Santa Antonieta mine in the district of Cartagena–La Union Mining, Murcia, SE Spain, with an area of 1.4 ha (Fig. 1). The climate is Mediterranean semiarid with a mean annual rainfall of 275 mm and an average yearly temperature of 18 °C (Martínez-Martínez et al., 2019).

Tailings are classified as Espodic Technosol (IUSS, 2014) and characterized by absence of vegetation, sandy loam texture, extreme acidity, low content of nutrients and organic matter and high metal(loid)s concentrations. Major metals associated with this area include Zn (829–5899 mg kg^−1^), Pb (1246–2048 mg kg^−1^), Cu (41–124 mg kg^−1^) and Cd(0.73–6.65 mg kg^−1^) (Martínez-Martínez et al., 2019).

### Experimental design and soil sampling

The tailings pond surface was tilled, levelled and a drainage system was established to prevent the formation of runoff and flooded zones. In order to enhance soil nutrients and soil organic matter, neutralize acidity, reduce the availability of potentially toxic elements, facilitate vegetation establishment and improve soil structure, marble waste (CaCO_3_), pig slurry and pig manure were applied as amendments (July 2011). Amendments were mechanically added and then mixed to a depth of 0.5 m of surface soil. Table 1 shows the characteristics of the soil amendments. In accordance with the guidelines in Council Directive 91/676/EEC and RD 261/1996 to avoid the salinity and contamination by nitrates, pig slurry was applied at a rate of 1.7, 2.6 Lm^−1^, respectively, followed by 7 kgm^−1^ of pig manure. In addition, 6.7 kgm^−2^ of marble waste, as calculated by Sobek et al. (1978) method, was applied to neutralize the potential acidity generated from the oxidation of mineral sulfides present in the soil when rainfall event take place in the area, which generate acid mine drainage (AMD).

**Table 1:** Main characteristics of the marble waste, pig manure and pig slurry used (Martinez-Martinez et al., 2019)

Properties	Marble waste	Pig manure	Pig slurry
pH	8.0	9.1	7.8
Electrical conductivity (dS m^−1^)	2.2	10.2	39.1
Moisture (%)	1.0	10.0	96.0
Total nitrogen (g L^−1^)	^–^	13.6	4.8
NH_4_^+ −^ N (g L^−1^)	^–^	^–^	4.5
Total organic carbon (g L^−1^)	^–^	171	17.8
C/N	^–^	12.5	4.3
Calcium carbonate (%)	99	^–^	^–^
Potassium (mg L^−1^)	59	15.7	1059
Sodium (mg L^−1^)	69	4280	459
Magnesium (mg L^−1^)	347	802	14.4
Calcium (mg L^−1^)	2190	855	249
Cu (mg kg^−1^/mg L^−1^)	0.36	157	19.3
Zn (mg kg^−1^/mg L^−1^)	0.26	732	28.0
Available phosphorus (mg kg^−1^/mg L^−1^)	0.01	9.6	623

Eight months after amendment application (March 2012), the following native species was planted: *Atriplex halimus* L., *Cynodon dactylon* (L.) Pers*.*, *Diplotaxis erucoides (*L.) DC*.*, *Lobularia maritima* (L.) Desv., *Oxalis pes-caprae* L., *Phagnalon saxatile* (L.) Cass*.*, *Piptatherum miliaceum* (L.) Coss*.*, *Senna occidentalis* (L.) Link*.* and *Stipa tenacissima* L.

After 3 years of the amendments application and planting (March 2015), a different vegetation development was observed on the surface of the tailing pond; therefore, in order to identify what factors could cause this difference, four plots (10 m × 10 m) with different vegetation coverage (VC) were selected: plot 1 with VC of 85%, plot 2 with VC of 45%, plot 3 with VC of 20% and plot 4 with VC of 0%. In addition, a fifth plot of mining pond with no vegetation nor amendment application was chosen to be used as control plot (Fig. 1), which represents the characteristic of the tailing pond before rehabilitation. In each plot, random soil sampling (0–50 cm) was carried out with five replications.

In order to evaluate the biodiversity and heterogeneity of plant communities, all plant species were identified and then species richness and Shannon–Wiener index (H) estimated (Shannon & Weaver, 1963).

### Laboratory analysis

Soil electrical conductivity (EC) and pH were determined in ratios of 1:5 and 1:2.5 sample to deionized water suspension, respectively. Particle size distribution was done by Robinson pipette methodology. Available phosphorus (P) was determined using the Olsen method (Watanabe & Olsen, 1965). Total nitrogen (TN), total sulfur (S) and total organic carbon (TOC) were examined by an elemental CHNS analyzer (EA-1108, Carlo Erba). Aggregate stability and calcium carbonate equivalent (CCE) were measured using (USDA-NRCS, 2004) methods. Total porosity (P) was calculated using real and bulk densities.

Bioavailable metal concentrations were determined with 0.01 M CaCl_2_ (1:10 soil–extractant ratio) (Pueyo et al., 2004). Water-soluble metals were examined using a 1:5 soil-deionized water ratio (Ernst, 1996). Pseudo-total metals (Pb, Cu, Zn and Cd) were evaluated using HNO_3_/HClO_4_ digestion at 210° C for 90 min (Risser & Baker, 2018). Sequential extraction procedure developed by Tessier et al. (1979) and modified by Li et al. (1995) was used for determining the chemical speciation of metals in soil. The following fractions were obtained: (1) exchangeable (using 0.5 M MgCl_2_), (2) bound to carbonate and specifically adsorbed (using 1 M NaOAc), (3) reducible and bound to Fe/Mn-oxides (using 0.04 M NH_2_OHHCl), (4) oxidizable and bound to sulfides and organic matter (using 0.02 M HNO_3_, H_2_O_2_ (30%) and 3.2 M NH_4_OAc in 20% (v/v) HNO_3_) and (5) residual phase (using HNO_3_ (65% w/w) and HF (40% w/w)). Measurements of the concentrations of metals were measured using an atomic absorption spectrophotometer (AAnalyst 800, Perkin–Elmer). Certified reference materials BAM-U110 (Federal Institute for Materials Research and Testing) and reagent blanks were used to verify the quality of analyses.

### Data analysis

Kolmogorov–Smirnov test was applied to ensure the data fit a normal distribution. Some data did not follow the normal distribution and log transformation was done. All data obtained were subjected to analysis of variance (ANOVA) using SAS statistical program (ver.9.1) and differences among traits were compared by the least significant difference (LSD) test (p ≤ 0.05).

The relationship among soil bioavailable metals and physicochemical characteristics, and grouping them into a few components were studied by principal component analysis (PCA). In this case, varimax rotation was applied to make the patterns of loadings more pronounced while also facilitating the interpretation of the results (Field, 2009). To group existing soil samples based on their physicochemical properties and to have a suitable visual representation of the achieved results, k-means algorithm was adapted for soil data clustering. K-means algorithm form clusters with similar data samples based on the square Euclidean distance method (Hartigan & Wong, 1979; Hot & Popović-Bugarin, 2016). These analyses were performed using R 3.2.5 (R Development Core Team, 2015) and RStudio (version 0.99.903) (RStudio, 2021).

## Results and discussion

### Plant growth

As shown in Table 2, different plant species were established unevenly in the studied plots. A total of 152 individuals of 9 plant species were recorded growing in three plots (plots 1, 2 and 3) in the tailing pond. The most common species was *P. miliaceum* with the abundance that varied between 10 and 34 individuals among the revegetated plots.

**Table 2:** Richness, Shannon–Weaver index, vegetation cover and plant species found in each plot

Plant/plot	Plot 1	Plot 2	Plot 3	Plot 4
*Atriplex halimus L*	34	5	0	0
*Cynodon dactylon (L.) Pers*	0	6	3	0
*Diplotaxis erucoides (L.) DC*	0	4	0	0
*Lobularia maritima (L.) Desv*	8	0	0	0
*Oxalis pes-caprae L*	2	1	0	0
*Phagnalon saxatile (L.) Cass*	6	2	0	0
*Piptatherum miliaceum (L.) Coss*	24	10	34	0
*Senna occidentalis (L.) Link*	1	0	0	0
*Stipa tenacissima L*	8	4	0	0
Richness	7	7	2	0
Shannon–Weaver index	1.5	1.76	0.28	0
Vegetation cover (%)	85	45	20	0

Vegetation cover plays a key role in enhancing nutrient inputs to the ecosystem, increasing soil organic matter (SOM), protecting soil surface from raindrop splashing, increasing water holding capacity and promoting soil aggregate stability. Although organic amendments and marble waste were applied homogeneously in the whole tailing pond, plants grew differently on the surface of the tailing ponds, as showed in the different values of vegetation cover observed in each plot (Table 2). Due to the similar parent material, remediation practices and climate condition of the studied tailings ponds, it would be likely that apart from these factors, other driving parameters such as topographic position of the region might also contribute to the vegetation development diversity.

Biodiversity evolution is an important parameter to be monitored in the aided phytostabilization program. Increasing biodiversity such as plant species is a good indicator of the durability and quality of the immobilization of trace elements (Vangronsveld et al., 2009). The Shannon–Weaver biodiversity indexes calculated for the plots 1, 2 and 3 were 1.5, 1.76 and 0.28, respectively, whereas their associated richness was in the order of 7, 7 and 2 (Table 2). *P. miliaceum,* dominated in plots 2 and 3, showed a good adaptation to the soil conditions of the plots, except for the plot 4. This grass and *A. halimus L.*, major species in plot 1, were the dominant species of the vegetation community in the tailing pond (Table 2). This finding was in agreement with the results reported by Martínez-Martínez et al. (2019) in tailings ponds of the same mining area. Conversely, only a single individual of *S. occidentalis* was observed throughout the mining district, colonization by this weed was only limited to the plot 1.

### Effect of amendments and phytostabilization on soil properties

The main physicochemical properties of the soils are reported in Table 3. Prior to the amendment application, pH of the tailing pond was acidic (pH = 2.9) which tended to increase toward neutral by aided phytostabilization. Low pH in these waste materials was due to the oxidation of pyritic minerals. The generation of hydronium (H_3_O^+^) in the oxidation process along with the presence of Zn and Cu sulfates and sulfuric acid keeps the pH of the soil very low (Martinez-Martinez et al., 2013). The presence of marble waste that generated carbonates resulted in pH increment, except for plot 4. Replacement of Al^+3^ of the exchangeable complexes by Mg^+2^ and Ca^+2^ along with a larger surface area for H^+^ adsorption created by small particles of marble waste promoted the neutralization of the acidity (Tozsin et al., 2015). EC values were significantly lower (*p* ≤ 0.05) in treated plots than in bare soils (plot 4 and control), without significant differences among vegetated parts. Marble waste application resulted in a significant reduction in soil EC. The reaction of sulfates from sulfides oxidation with Ca^2+^ from CaCO_3_ led to forming stable precipitates and reducing the sulfates concentration in soil solution. In addition, pH increments might also favor the precipitation of some soil solution ions, thus reducing soil EC (Brallier et al., 1996).

**Table 3:** Physicochemical properties and metal concentrations (mg kg^−1^) of soil samples from the studied tailing pond, mean (standard deviation)

Soil properties	Plot 1	Plot 2	Plot 3	Plot 4	Control*
pH	7.1(0.6)a	5.8(1.4)ab	4.7(1.5)b	3.3(0.3)c	2.4(0.3)c
EC (dS m^−1^)	3.0(0.1)b	4.0(0.4)b	4.4(0.7)b	7.4(0.8)a	6.9(2.2)a
CCE (%)	2.9(2.3)a	1.5(0.6)ab	1.3(0.6)b	0.5(0.0)c	0.5(0.1)c
OC (g kg^−1^)	1.0(0.5)a	0.4(0.1)b	0.3(0.1)bc	0.3(0.2)bc	0.2(0.1)c
TN (g kg^−1^)	0.1(0.0)a	0.0(0.0)b	0.1(0.0)a	0.1(0.1)a	0.0(0.0)b
TS	0.4(0.2)d	0.5(0.2)cd	0.8(0.3)c	1.5(0.2)b	2.8(0.3)a
P	31.1(9.2)a	10.0(1.2)b	8.6(1.1)b	7.7(1.1)b	8.8(1.7)b
Clay (%)	9.2(1.1)b	7.8(0.4)c	7.8(0.9)c	10.8(1.4)a	9.4(1.1)b
Silt (%)	17.3(2.4)a	13.2(1.3)b	11.1(0.8)cd	9.6(0.9)d	12.7(1.3)bc
Sand (%)	73.5(1.6)c	79.1(1.4)ab	81.1(1.1)a	79.6(2.1)ab	77.8(1.7)b
Aggregate stability	15.5(1.7)b	17.8(2.4)b	18.4(3.7)b	17.3(2.0)b	21.5(2.7)a
Porosity	57.8(2.1)c	67.4(3.9)a	63.1(1.3)b	64.7(1.6)b	64.7(0.5)b
Total Pb	1298(72)b	1567(181)a	1753(174)a	1286(115)b	1176(208)b
Total Cu	57(3.9)c	59(2.3)c	76(20)b	74(3.3)b	94(10)a
Total Zn	4120(940)a	2738(236)b	2268(276)b	3524(513)a	2364(234)b
Total Cd	7.8(2.0)a	4.7(0.6)b	4.3(0.5)b	6.4(1.4)a	4.5(0.4)b
Bioavailable Pb	49(20)a	12.6(3.2)b	0.7(0.1)c	8.8(1.5)b	0.6(0.2)c
Bioavailable Cu	1.3(0.3)b	0.9(0.24)b	1.0(0.1)b	3.3(0.7)a	3.2(0.2)a
Bioavailable Zn	83(21)c	39.8(20)c	60.9(8)c	823(79)a	290.6(20)b
Bioavailable Cd	0.5(0.0)c	0.3(0.1)c	0.4(0.1)c	2.7(0.6)a	1.0(0.1)b
Soluble Pb	0.7(0.1)a	0.1(0.0)b	0.0(0.0)c	0.1(0.0)b	0.0(0.0)c
Soluble Cu	0.1(0.0)c	0.1(0.0)c	0.1(0.0)c	1.7(0.3)b	2.2(0.2)
Soluble Zn	1.1(0.2)d	0.7(0.1)d	50.8(9)c	565(47)a	214.7(28)b
Soluble Cd	0.1(0.0)d	0.1(0.0)d	0.3(0.1)d	2.1(0.1)a	0.8(0.1)b

Rows with different letters indicate significant differences at *p* < 0.05

*EC* electrical conductivity, *CCE* calcium carbonate equivalent, *OC* organic carbon, *TN* total nitrogen, *TS* total sulfur, *P* available phosphorus

Untreated tailing

After application of marble waste, CCE content in soil enhanced significantly by up to 2.9% in plot 1. Aided phytostabilization had a significant effect on OC values in plots 1 and 2 compared to the other plots (Table 3). Such increment could not be only related to the effect of organic amendments but also attributed to the development of vegetation cover. Some authors concluded that the application of pig slurry and pig manure in tailing ponds improve soil structure and promote microbial activity and population (Kabas et al., 2012; Zornoza et al., 2013).

The amount of TN showed a significant increase in treated plots compared to the control, except for plot 2, whereas for the rest of samples the differences were not significant. After amendment application, the highest available *p* value was measured in the soil of plot 1 compared to the control (Table 3). Pig slurry and manure as a source of slow-release nutrients are rich in nutrients essential for vegetation such as N and P. The low mineralization rate of these organic amendments favored the increase of P and N in soil (Peu et al., 2007).

Treated soils exhibited a significant reduction in TS when compared to the control samples, owing to the sulfur utilization by plants, particularly in sulfate forms (Kabas et al., 2012) and also oxidation of total sulfur in surface after ploughing. Exposure of sulfides to oxidizing conditions results in the release of sulfate anions and metal cations. The reaction of sulfates with calcium generates stable precipitates and thus decreases the concentration of sulfates in soil. pH increment might also cause in the precipitation of ions in the soil solution (Fernando et al., 2018a, 2018b).

The amounts of clay, silt and sand were found to vary from 7.8 to 10.8, 9.6 to 17.3, and 73.5 to 81.1%, respectively, that exhibited no significant difference after the application of amendments. All the soils had a sandy loam texture. Aggregate stability in treated plots reduced significantly in comparison with the control. However, Kabas et al. (2014) reported an increase in aggregate stability in the tailings ponds treated with MW, PS and MW + PS after 6 and 12 months of sampling. They attributed the aggregate stability increment to the cohesive forces derived from the interactions between root zone organisms of pioneer species and plants. The porosity variation in the soil of the studied plots compared to the control samples followed no clear pattern, with no significant differences (Table 3).

Our findings also indicated a significant positive correlation between the percentage of vegetation cover and the values of pH, CCE, OC, P and silt, while the contents of EC, TS and sand decreased with increasing the vegetation cover percentage (Table 3). Such positive correlation between pH, total organic carbon, available *p* and vegetation development was also observed by previous investigators (Kabas et al., 2012). The application of soil amendments contributed to the soil quality and fertility improvement facilitating a higher colonization of vegetation (Acosta et al., 2018).

As the general conditions of the studied plots, including parent material and the management of phytoremediation process, have been similar, the difference in the percentage of vegetation cover among plots could be attributed to different soil characteristics. For instance, plot 4 remained practically bare and exhibited differences for the soil properties, including pH, EC, TS and clay compared to the other vegetated plots. Many environmental processes (e.g., evapotranspiration, rainfall and runoff) and physical factors (e.g., topography) operating at various intensities over different scales give rise to a complex spatial variability of hydrological processes and thus different patterns of soil water content, soil properties and vegetation on and within a landscape (Biswas, 2019). Therefore, the different soil and vegetation characteristics of plot 4 might be attributed to the position of this plot in the tailing pond, which location is closest to non-rehabilitated areas and a slightly lower elevation than these areas, therefore on some occasions, it can receive runoff water from these non-rehabilitated areas. Inherent acidic conditions of this plot as well as accumulation of higher amounts of soluble and available metals, and salts from adjacent areas have made it toxic and undesirable to plant growth and prevented the vegetation establishment in such soil. Therefore, both the topographic position and amendment application could affect the soil properties and thus the vegetation cover of the studied plots and to somewhat created different variation patterns among the remediated groups.

### Effect of aided phytostabilization on the availability of metals

The total metal concentrations in this tailing pond (Pb: 1176–1753, Cu: 57–94, Zn: 2268–4120 and Cd: 4.3–7.8 mg kg^−1^) were lower than those in other contaminated mining districts under similar climatic conditions (De la Fuente et al., 2014). After the application of amendments and promotion of vegetation development, the values of total metals indicated a different trend in each plot as compared to the control, except for Cu that reduced in treated areas (Table 3). The purpose of aided phytostabilization technique is not to decontaminate the area, but to immobilize soil metals, reduce their availability to living organisms and thus minimize the risk to the environment and human health (Radziemska et al., 2018). In this regard, no significant correlations were found between total concentrations of metals and vegetation cover percentage in treated plots, except for Cu, which confirmed this fact. Total Cu value indicated a negative correlation with vegetation cover percentage in the study area, probably because of copper is a micronutrient for plants and it is absorbed by them.

Regardless of the plot 4, available and soluble fractions of Cu, Zn and Cd were higher than the rest of studied plots (Table 3). This increase is probably attributed to the direct effect of soil pH. Contrarily, in plots 1, 2 and 3 the presence of carbonates and organic matter from the amendments promoted the formation of metal carbonates and complexation by organic matter (Alvarenga et al., 2008; Zornoza et al., 2013)and therefore a decrease of available and soluble metals. This result is in agreement with that reported by previous researchers studied the contaminated mining areas (Liu et al., 2009; Shahrokh et al., 2022). The use of pig slurry and manure containing a high quantity of nutrients have positive impacts on soil properties, seed germination and reclamation strategies (Pardo et al., 2011). Acosta et al. (2018) also concluded that immobilization by carbonates provided by marble waste was required to decrease the availability of Zn.

As mentioned earlier, *P. miliaceum* and *A. halimus* are the most dominant species developed in the studied soils. Martínez-Martínez et al. (2019) demonstrated that *P. miliaceum* was successful in accumulation of large amounts of Zn and Cu in its roots and have a translocation factor (TF) < 1 and a bioaccumulation coefficient (BCF) > 1. In addition, *A. halimus* and *P. saxatile* were the species with high concentrations of Zn and Cd in their leaves (Acosta et al., 2018). As these species were the few species were able to survive and grow under the acidic conditions of the tailing pond (Shahrokh et al., 2022), it is suggested monitoring the long-term growth and effectiveness of them as some candidates for phytostabilization of mining districts.

Available and soluble Pb in treated waste increased as compared to the control and the highest values of this metal were measured in plot 1. Krol et al. (2020) found a strong dependence of potentially toxic elements (Ni, Cd, Cu, Pb, Zn and Cr) leaching on the pH from metallurgical slag. They claimed that heavy metals concentrations decreased with an increase in the pH of the solution, except for Pb. Pb represented an upward release trend under alkaline conditions. In addition, Gustafsson et al. (2011) and Wang et al. (2018) also reported that Pb adsorption on organic matter is more prevalent at soil pH < 6. As soil pH in plot 1 is 7.1, it could be expected that Pb bioavailability in this plot is higher than that in other plots.

Plot 4 showed the highest concentrations of available and soluble Cu, Zn and Cd, this is likely due to the position of this area in the tailing pond (Kabas et al., 2012). During the intense precipitations, this lower elevation respect to not treated waste (control area) became waterlogged receiving runoff from this part of the tailing pond (Acosta et al., 2018). Due to the acidic nature of these waters, metal transportation from other contaminated adjacent areas as well as dried plants and plants residues enriched in potentially toxic elements, in fact, the soil of plot 4 obtained the highest concentrations of bioavailable metals among the studied plots.

**Fig. 2:**
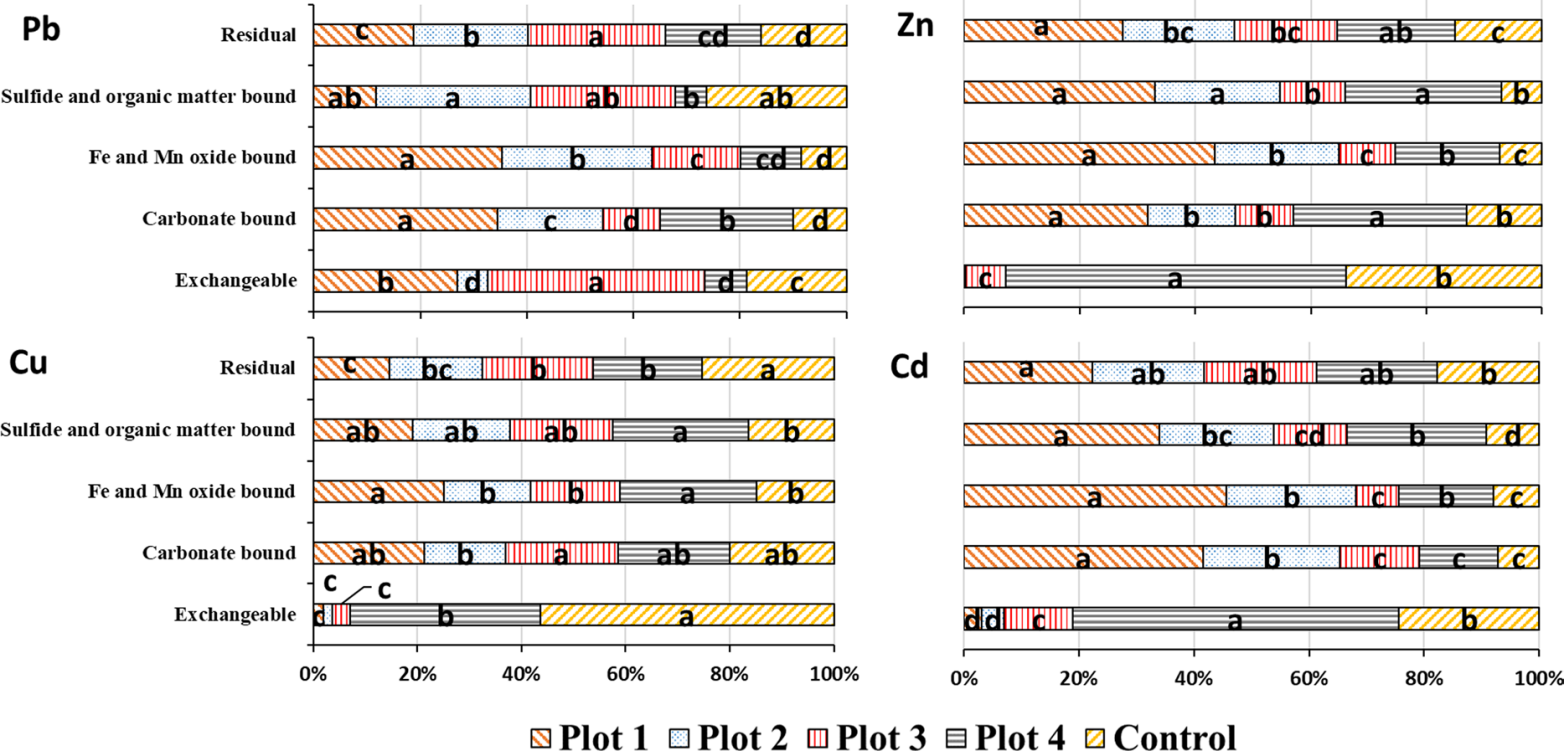
Distribution of Pb, Zn, Cu and Cd in the solid phases of studied plots

The results also revealed that the concentration of bioavailable Cu, Zn and Cd showed a negative significant correlation with the percentage of vegetation cover, whereas the correlation between bioavailable Pb and vegetation cover percentage was significantly positive. This finding is consistent with our previous achievements. Shahrokh et al. (2022) and Kabas et al., 2014 also reported the high accumulation of metals in established plants tissues in mining wastes (BF > 1). It is concluded that high soil salinity and soluble concentrations of Mg, K and Na and the soil total concentrations of metals contribute to the metals accumulation in *A. halimus* leaves (Shahrokh et al., 2022). Halophytic plants accumulate cations in their tissues to regulate the internal osmotic pressure. In this line, halophytes have been proposed as desirable alternatives for metals removal and desalinization of saline and non-saline soils (Manousaki & Kalogerakis, 2009). Several investigators found that a soil salinity increment increases the metals soluble concentration by the formation of soluble inorganic complexes (Abbaspour et al., 2008; Ghallab & Usman, 2007). The metals retained in the five solid phases for studied plots can be seen in Fig. 2. For Cu, Zn and Cd, exchangeable fraction of the soils in plots 1, 2 and 3 showed the lowest value, while that in the soils of plot 4 and control was the most dominant fraction. This result confirms the approach of phytostabilization regarding declining the bioavailability of metals in contaminated regions over time. Acosta et al. (2018) also evaluated the suitability of aided phytostabilization on this tailing pond and reported that this technique decreased the mobility of metals mainly Pb, Zn and Cd. Furthermore, exchangeable fraction of metals in plot 3 showed a similar behavior to that in the control samples, probably owing to the lack of vegetation cover in this area.

The exchangeable fraction of Zn, Cu and Cd significantly decreased with increasing the percentage of vegetation cover in treated soils. The highest and the lowest values of exchangeable phase were obtained in the plots 4 and 1, respectively. For Pb in control soil, the fraction with the highest concentrations and percentages was the oxidizable phase followed by exchangeable form. A significant high content of Pb bound to the oxidizable phase in this plot is likely attributed to the high content of sulfides in the surface layer. Similar result has been also reported on the tailing pond contaminated with Pb by Martínez-Martínez et al. (2013). After the application of amendments and vegetation establishment, the amount of exchangeable Pb in the soil decreased in the order: plot 3 > plot 1 > plot 2 = plot 4. Clearly, sequential extraction data of Pb did not follow a similar pattern to those of other metals.

### Relations among soil properties and available metals under aided phytostabilization

**Fig. 3:**
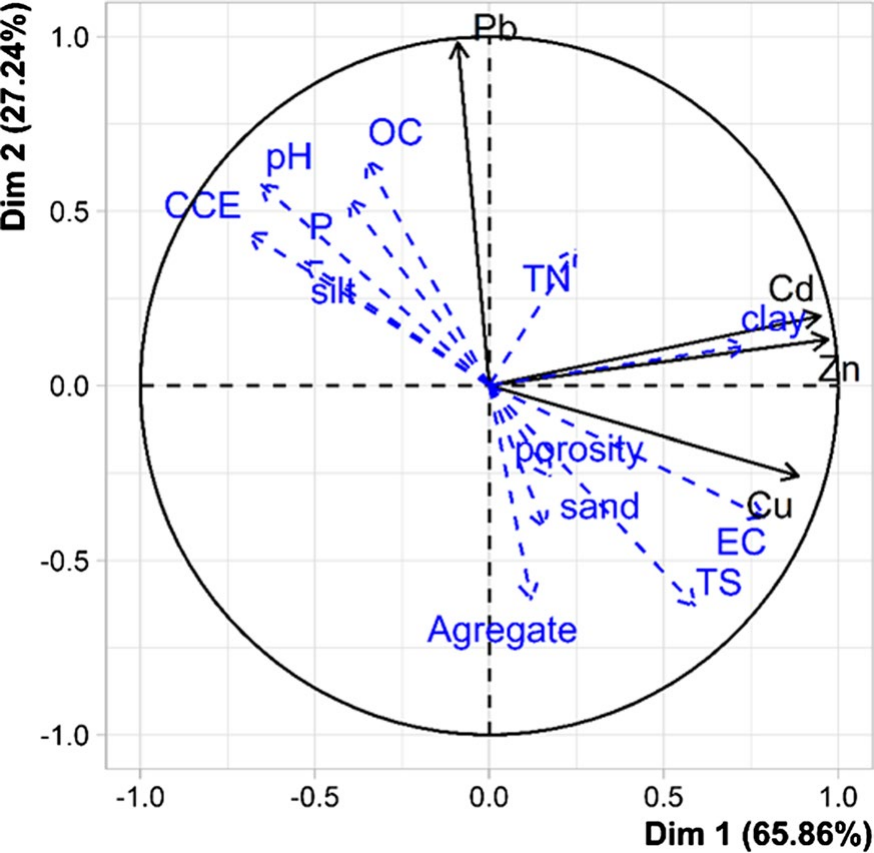
Biplot drawn based on the first (PC1) and second (PC2) components obtained from principal component analysis. *OC* organic carbon, *EC* electrical conductivity, *TS* total sulfur, CCE; calcium carbonate equivalent, *P* phosphorus

The PCA provided a model in which the first two PCs accounted for 93% of the total variance among the studied soils (Fig. 3). PC1 exhibited high to moderate positive loadings on bioavailable Cu, Zn and Cd, clay and EC, whereas pH and CCE were negatively associated in PC1. This finding suggests that an increase in CCE resulted in pH increment and promoted a decrease in availability of soil metals. Martínez-Martínez et al. (2019) found a negative correlation between pH and available metals in the tailing pond. They reported that pH increment could increase the activity of some enzymes in soil, which in turn decreased the bioavailability of Cu, Zn and Pb. Khanmirzaie et al. (2013) investigated the relationship between different forms of Cd in soil and uptake by wheat and reported that there was a significant relationship between bioavailable Cd and EC.

PC2 explained 27% of total variance showed high positive loading on Pb and OC, whereas it was negatively related to total sulfur (Fig. 3). Sulfide oxidation in soil results in the formation of iron oxides and hydroxides, which could precipitate As and reduce its availability (Sun et al., 2012). The decrease in metal availability could happen in soil owing to the formation of stable complexes with organic matter (Yolcubal & Akyol, 2008) and the precipitation with carbonates during aided phytostabilization process.

**Fig. 4:**
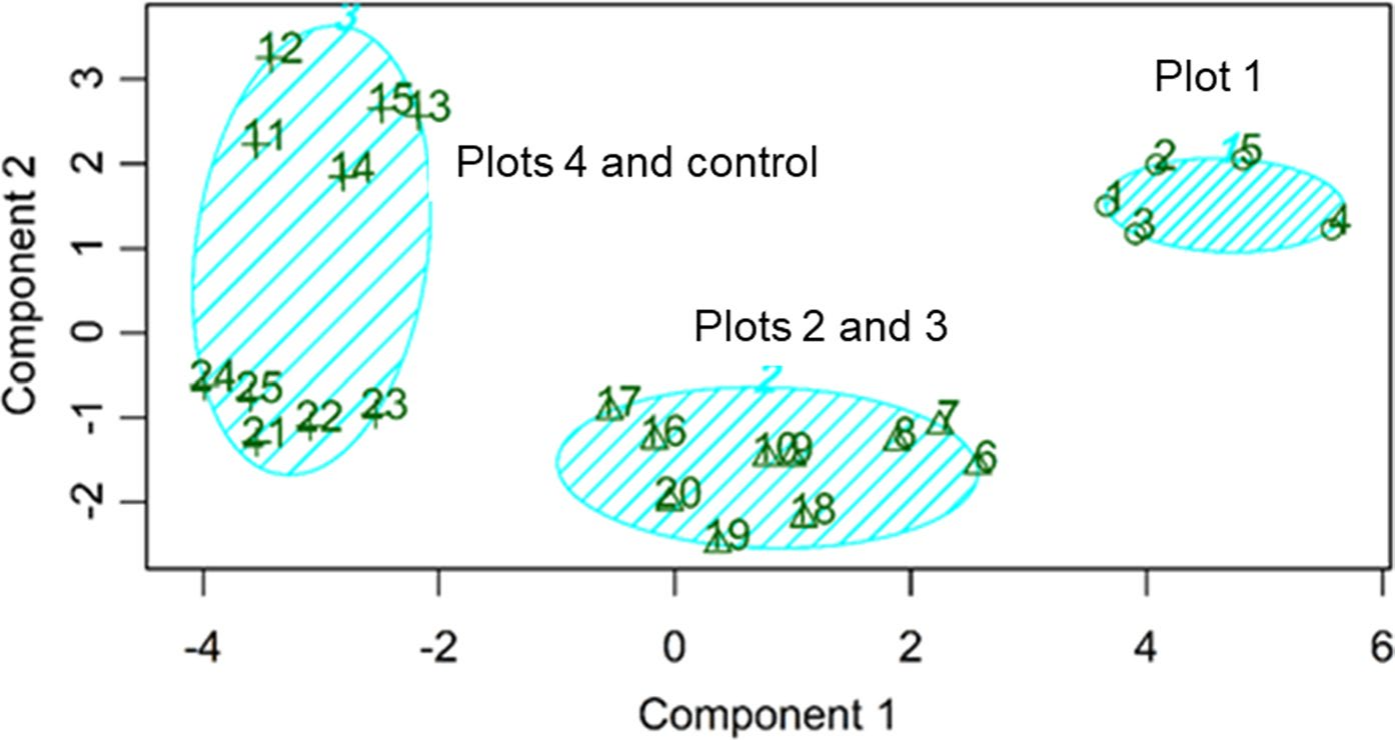
K-means clusters of studied plots

The cluster analysis of the samples shows the existence of three main groups (Fig. 4). The first group consists of the samples from plot 1, second group includes samples from the plots 2 and 3, and the third one comprises the samples of the plots 4 and control. This is in good agreement with the previous findings regarding the major effect of vegetation cover on soil properties particularly on bioavailable metals. Plot 1 with the highest percentage of vegetation cover presented the most significant impact on the characteristics of soils. Plots 2 and 3 with almost similar vegetation cover percentages showed a similar behavior of influencing the soil. In addition, our findings indicated that plot 4 due to the lack of vegetation cover followed the trend of control plot in altering the heavy metals fractions.

## Conclusions

Aided phytostabilization including the application of amendments along with vegetation of native species could significantly improve the chemical conditions of an acidic tailing pond, which resulted in the increment of vegetation cover percentage, richness and biodiversity index. Marble waste, pig slurry and manure were efficient in decreasing soil EC, TS and bioavailability of metals including Zn, Pb, Cu and Cd, while pH, CaCO_3_, OC and TN were increased after application of these amendments. Besides the amendments, plots location was another factor that affected the soil properties and phytostabilization efficiency in the studied plots. Plot 4 due to its lower topography situation in the tailing pond receiving runoff water from the non-rehabilitated areas contained higher concentrations of potentially toxic elements and remained bare. Therefore, this plot exhibited a trend similar to the control regarding the variation of soil characteristics after the reclamation strategy, which was confirmed by the results of sequential extraction analysis. Furthermore, pH, CCE, OC, TN and P were positively correlated with the percentage of vegetation cover, while a negative correlation was found between EC and TS and vegetation cover percentage. *A. halimus*, *P. miliaceum* and *P. saxatile* were the most dominant species stablished in the treated plots.

This study showed that in relation to the establishment and growth of plants and consequently the efficiency of phytostabilization, even a slight change in slope can be a very critical factor that must be considered. Many researchers and managers ignore the examination of micro-topographic features as effective factors in phytoremediation process, and must be taking in to account in remediation projects.

## Data Availability

The authors confirm that the data supporting the findings of this study are available within the article**.**

## Declarations

### Animal research

No application.
